# The spillover effect of after-hours electronic communication on nurses’ cyberloafing: the mediating role of psychological contract breach

**DOI:** 10.1186/s12912-023-01525-0

**Published:** 2023-09-27

**Authors:** Na Zhang, Xiaoyun Liu, Jingjing Li, Zhen Xu

**Affiliations:** 1 School of Economics and Management, Beijing Information Science & Technology University, Beijing, China; 2 College of Economics and Management, Beijing University of Technology, Beijing, China; 3 Medical College, Hebei University of Engineering, Handan, China

**Keywords:** After-hours electronic communication, Psychological contract breach, Cyberloafing, Spillover effect

## Abstract

**Background:**

Considerable research has investigated the influencing factors of cyberloafing in the workplace. However, few studies have focused on the antecedents in non-work fields, especially for nurses. According to the effort-reward imbalance theory, this study aims to explore the spillover effect of after-hours electronic communication on nurses’ cyberloafing, and the mediating role of psychological contract breach.

**Methods:**

A total of 282 nurses completed the online survey. PROCESS macro for SPSS was used to test how after-hour electronic communication affect nurses’ cyberloafing.

**Results:**

After-hours electronic communication has a significant positive impact on nurses’ cyberloafing, and psychological contract breach plays a mediating role in the relationship.

**Conclusion:**

Psychological contract breach was the linchpin linking after-hours electronic communication to nurses’ cyberloafing in workplace. This study provides a guide for healthcare organizations to reduce or manage inappropriate telework arrangements and strengthen nurses’ psychological contracts.

## Background

Since information and communication technologies (ICT) are developing rapidly in today’s world, many organizations are committed to digital transformation [1]. In health care industry, ICT is widely used in the field of nursing [2]. When nurses provide medical services, they make extensive use of Internet tools to record patient data and access patient information [3]. The increasing use of ICT also facilitates the sharing of information and provides patients with access to quality health resources [4].

However, a phenomenon (that is, cyberloafing) resulting from continually increasing human-computer interaction emerged. Compared with the traditional sabotage, cyberloafing has the characteristics of high imperceptibility (employer is unaware of how employees utilize the Internet resources of the organization) and high deceptive (employees use the Internet to pretend to be active) [5]. In nursing, cyberloafing means even greater losses. Cyberloafing can distract nurses and increase the possibility of operational errors, leading to substandard care practices [6]. Previous surveys have shown that many employees spend a significant amount of their work time on cyberloafing [7], which wasted a lot of time resources and brought unnecessary losses for organizations [8, 9]. For any organizations, this issue has posed a serious management challenge. This is an even more important issue in the medical field [10].

Cyberloafing, as a new form of workplace deviance, is defined as “any voluntary act of employees” [11] using ICT during office hours to access social media and other websites for personal and nonwork-related activities or benefits [1]. Numerous studies have shown that cyberloafing not only reduces employees’ work engagement, leading to lower productivity [12], but also occupies network resources and increases the risks of organizational information insecurity [13], which results in substantial costs to organizations [14]. Therefore, further research is required to identify the underlying mechanism and influencing factors of nurses’ cyberloafing to effectively manage.

Over the past two decades, most of the studies on cyberloafing have focused on the antecedents in the workplace. Previous researches have indicated that the organizational environment [15, 16], supervisor attitude [11, 17], and peer co-worker behavior [18] may affect employees’ emotions and attitudes, which then lead to the occurrence of employee cyberloafing. However, the understanding of factors in the nonwork context as antecedent variables is limited.

Work field and nonwork field are the two places for employees’ activities. The permeability of the border between the two field allows elements such as information, resources, and emotions to flow from one field to another [19], resulting in spillover effects [20]. In other words, employees’ psychological perception and emotional experience in one field will affect their psychology and behavior in another field. For example, previous studies have shown that if employees obtain sufficient resources from places other than the workplace, they will also feel that they are thriving at work [21]. Therefore, in order to make a profound study the inducing factors of cyberloafing, it is necessary to explore the factors of employee cyberloafing in the nonwork context and detect the spillover effect from the non-workplace to the workplace. In consideration of this undeveloped area, we will focus on solving this problem by analyzing how factors in the nonwork context influence nurses’ cyberloafing.

After-hours electronic communication (AEC), as the side of ICT, has also raised concerns. The growth in the elderly population and the emphasis on health make the shortage of nurses, who can only cope with the heavy workloads by reducing their non-working time [22]. As the person who has the closest contact with patients, nurses need to be prepared to always serve patients, even during non-working time. AEC refers to employees logging back into work (or never logging off) to address work-related electronic communications after normal work hours [23]. Although AEC deals with work related matters, it is still a nonwork context factor because it crosses both the physical boundary (AEC usually occurs outside the workplace, such as at home) and the time boundary (AEC occurs after work hours) between work field and nonwork field [24], and therefore employees are not usually paid accordingly [25]. From the perspective of employees, even if they are not in the office, they need to respond to many unexpected work assignments, which makes them feel compelled to deal with multiple tasks at a same time outside of normal working hours [26]. Employees’ attitudes and behaviors will be affected [27].

Existing research have demonstrated that AEC has a significant relationship with employees’ attitudes and behaviors in the work field. For example, it reduces employees’ work engagement [28, 29], leads to job burnout [30], and leads employees to engage in counterproductive work behaviors [31]. Overuse of technical tools that are critical to multitasking may also provide opportunities for employees to frequently use social media sites [32]. In other words, when employees are faced with frequent electronic communication during off-duty time, they are likely to choose cyberloafing as an answer to the pressure caused by AEC [26, 33]. Based on the study, we consider the positive correlation between AEC and nurses’ cyberloafing.

The spillover effect between work field and nonwork field occurs through employees’ psychological carryover [19]. In this study, we regard psychological contract breach as the key bridge connecting AEC and nurses’ cyberloafing because psychological contract breach reflects employees’ subjective perceptions of the organization, which should generate consequences for employees’ behavior [34]. Psychological contract breach is defined by Robinson as the cognition that one’s organization has failed to meet one or more obligations within one’s psychological contract in a manner commensurate with one’s contributions [35]. This is essentially caused by the unfair treatment of employees by organizations [36]. Therefore, we consider that psychological contract breach plays a mediating role in AEC and nurses’ cyberloafing.

## AEC and nurses’ cyberloafing

The balance between work field and nonwork field is an important appeal for employees [37]. AEC, as a form of work to nonwork field penetration, squeezes employees’ non-work time. According to the spillover, the negative experience caused by AEC can affect the performance of employees in the work field. First, AEC gives employees a sense of compulsion that employees are still responding to the organization outside of the office and office hours, which can easily cause dissatisfaction among employees [38]. Since the underlying meaning of AEC is that the organization expects employees to keep in touch to deal with the work at any time, employees are bound to be distracted by work tasks, even if the tasks has not yet arrived. Employees are not free to control their non-work activities [39], which can affect their work performance [27].

Second, employees cannot be removed from the work even during nonwork time [40], blurring the boundary between business and private life. Cyberloafing, an attempt to control the blurred border, is likely to be adopted by employees troubled by AEC [26, 41].

Third, when employees are engaged in AEC, they contribute their time and energy that should be used to deal with nonwork matters to work affairs. The resources needed to simultaneously accomplish nonwork affairs are not enough. Employees can only sacrifice extra their breaks for free. When employees are not well-rested, their work performance will be affected, resulting in negative attitudes and deviant work behaviors [42, 43]. The above reasoning leads to the following hypotheses:

### Hypothesis 1

AEC is positively related to nurses’ cyberloafing.

### The mediating effect of psychological contract breach

According to the effort-reward imbalance (ERI) theory, when the time, emerge and emotion invested by employees in work are not returned (reward-for-effort imbalance), it will lead to negative emotional and behavior consequences for individuals [44].

**Fig. 1:**
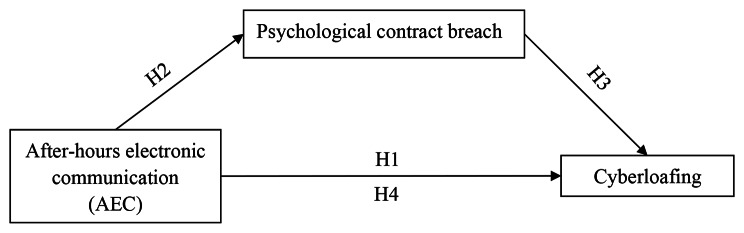
Research model

AEC, as a kind of invisible overtime and free digital labor behavior, does not provide corresponding rewards to employees, which will cause them to feel they are being treated unfairly. Fairness is crucial to maintaining employees’ psychological contracts [45]. Unfairness can lead to psychological contract breach. The negative mental state will lead to employees’ negative consequences in work field [46, 47]. Employees tend to reduce the level of effort or penetrate nonwork affairs into work field in order to balance their efforts and rewards [48]. That is, psychological contract breach plays a mediating role in AEC and nurses’ cyberloafing. Combining the above arguments, we propose the following hypothesis, as shown in Fig. 1:

#### Hypothesis 2

AEC is positively related to psychological contract breach.

#### Hypothesis 3

Psychological contract breach is positively related to nurses’ cyberloafing.

#### Hypothesis 4

The relationship between AEC and nurses’ cyberloafing is mediated by psychological contract breach.

## Methods

### Aim

The purpose of this study is to explore the influence mechanism of AEC (the imbalance between nurses’ efforts and rewards) on nurses’ cyberloafing (the way nurses try to balance efforts and rewards), and the mediating role of psychological contract breach.

### Data collection and participants

The current study adopted a cross-sectional research design to collect self-reported data from nurses in China. WeChat, one of the main software used by Chinese individuals to communicate, is the way we send out questionnaires. Each respondent was asked to snowball the questionnaire to their friends via WeChat. Data collection lasted from April to May 2022.

A total of 350 subjects completed the online survey and returned the questionnaire. After removing those invalid questionnaires, such as with duplicate IP addresses or submit in an unreasonably short period of time, 282 valid questionnaires were obtained, with a response rate of 80.57%. Among these samples, 67.4% of nurses surveyed are between the ages of 21 and 30. Most nurses were female, accounting for 94.3% of all respondents. 53.2% of the nurses surveyed worked for less than 5 years. Most nurses were junior college or higher (42.2% with a bachelor’s degree or high among them). Table 1 reflects a detailed description.

**Table 1:** Demographic characteristics (n = 282)

Demographics	Classification	Frequency	Percent	Cumulative Percent
Gender	Female	266	94.3	94.3
	Male	16	5.7	100.0
Age (years)	≤ 20	11	3.9	3.9
	21–30	190	67.4	71.3
	31–40	60	21.3	92.6
	> 40	21	7.4	100.0
Clinical tenure (years)	≤ 5	150	53.2	53.2
	6–10	67	23.8	77.0
	11–15	35	12.4	89.4
	16–20	13	4.6	94.0
	> 20	17	6.0	100.0
Education level	Certificate (technical school)	10	3.5	3.5
	Junior college	153	54.3	57.8
	Bachelor’s degree	117	41.5	99.3
	Master’s degree or above	2	0.7	100.0

### Measures

In order to ensure the reliability and validity of the measured variables, the measurement items were all using the existing scales in previous literatures. The scales we used are both developed in Chinese and originally originated in English. Since the survey-takers are Chinese nurses, we need to translate the English measures into Chinese to ensure the smooth progress of the investigation. Ultimately, we achieved the goal of no significant differences in language by recruiting professional translators.

#### AEC

The AEC scale consists of three dimensions was adapted by Chinese scholar Ma et al. [49, 50], which was proved to have good reliability and validity in China [51, 52]. Participants used a 5-point Likert scale to record the frequency of AEC during the previous month, ranging from 1 = never to 5 = frequently. The sample item for AEC was ‘How often are you contacted by work-related people during after-hours for handling work on the Internet’. Cronbach’s α for the current study was 0.76.

#### Psychological contract breach

Psychological contract breach scales were obtained from the 4-item scale that was developed by Robinson [53]. A sample item is ‘My organization has broken many of its promises to me even though I’ve upheld my side of the deal’. The items were recorded on a 5-point Likert-type scale ranging from 1 (strongly disagree) to 5 (strongly agree). Cronbach’s α coefficient of the scale was 0.82 in our research.

#### Cyberloafing

We used Lim’s scale [54] to measure cyberloafing, which included two dimensions: browsing activity (six items) and email activity (three items). Participants used a 5-point Likert scale to record how often they engaged in cyberloafing in the last month, ranging from 1 = never to 5 = frequently. Two examples were “Playing online games” and “Sending instant messages and chatting online.” Cronbach’s α for this scale was 0.90 in our study.

Reliability of all three scales in this study was well above the generally accepted threshold value of 0.70 [55, 56].

#### Control variables

Demographic variables (gender, age, clinical tenure, and education level) were used as control variables. We used dummy variables to measure them in this study. For gender: 0 for female and 1 for male. For age, 1 for 20 years or younger, 2 for 21–30 years old, 3 for 31–40 years old and 4 means over 40 years old. For clinical tenure, we categorized them into five categories: less than 20, 6–10 years, 11–15 years, 15–20 years, and more than 20 years. And we used the numbers 1 to 5 to represent them. For nurses’ education level, numbers 1 to 4 indicated certificate, junior college, bachelor’s degree, and master’s degree or above, respectively.

### Data analysis

We mainly used SPSS 23.0 and AMOS to analyze the collected data. First, to check the common method variance, Harman’s single-factor test was used. Second, we obtained the means and standard deviations of AEC, psychological contract breach and cyberloafing. Correlation values among these variables were obtained by using Pearson’s correlation analysis. Then, we used confirmatory factor analysis (CFA) to test measurement model. Finally, we used Hayes’s PROCESS macro for SPSS (Model 4) to test a simple mediation model with psychological contract breach as the mediator [57]. If the results obtained by using the recommended bootstrapping method with 5000 samples do not contain zero in the 95% confidence interval, the mediating effect was significant.

## Results

### Testing of common method variance

Self-report data can reduce the validity of results by common method variance. We tried to control the common method variance in the following two ways. We first considered procedural control method, starting with the questionnaire design and measurement, to minimize the common method variance. Following survey principles, we recruited participants who volunteered to answer the questionnaire and told them that they did not need to worry about their answers being leaked. We did not express variable names in the questionnaire, and we hid the research purpose. There are some reverse items in the questionnaire to avoid the consistency motivation of participants. And we used Data collectors to collect and input data. We effectively controlled the variance in the above ways. Besides, to determine whether the collected data would be affected by the common method variance, we examined the data by using Harman’s single-factor test. The generated PCA output revealed 16 distinct factors accounting 61.04% of the total variance. The first unrotated factor captured only 45.25% of the variance in data, less than 50% [58, 59]. Therefore, the common method variance of this study was within an acceptable range [60].

### Correlation analysis

Table 2 shows the means, standard deviations and correlations of AEC, psychological contract breach and cyberloafing (*n* = 282). The mean scores of the three variables were 3.37, 3.60 and 3.53 respectively. Their standard deviations were 0.938, 0.921 and 0.879. According to correlation analysis, we found that AEC is positively correlated with psychological contract breach (*r* = 0.503, *p* < 0.01) and cyberloafing (*r* = 0.572, *p* < 0.01). Psychological contract breach and cyberloafing also showed a positive correlation (*r* = 0.611, *p* < 0.01).

**Table 2:** Descriptive statistics and correlation matrix

Variables	Mean	SD	*1*	*2*	*3*	*4*	*5*	*6*	*7*
1. Gender	0.06	0.232							
2. Age (years)	2.32	0.668	-0.103						
3. Clinical tenure (years)	1.87	1.170	-0.096	0.852**					
4. Education level	2.39	0.570	-0.062	0.207**	0.122*				
5. AEC	3.37	0.938	-0.021	0.040	-0.063	0.070	(0.716)		
6. Psychological contract breach	3.60	0.921	0.040	0.078	-0.009	0.148*	0.503**	(0.738)	
7. Cyberloafing	3.53	0.879	0.027	0.022	-0.107	0.198**	0.572**	0.611**	(0.712)

Note(s): SD = Standard deviation, *p < 0.05(two-tailed), **p < 0.01(two-tailed). The numbers in parentheses on the main diagonal are the square roots of the average variance extracted (AVE).

### Measurement model

Before testing the hypotheses, we assessed the constructs’ convergent validity, discriminant validity and overall fit indices for the proposed model. Tables 2, 3 and 4 show these results. First, for convergent validity, we drew the following conclusions from Table 3: (1) the factor loadings values for all the items ranged from 0.586 to 0.814, higher than 0.40 [61]. (2) the AVE values for each construct were all above 0.50, and the CR values varied from 0.759 to 0.901, which were greater than the standard of 0.70, confirming convergent validity [62]. Second, we tested the discriminant validity by comparing the square root of AVE with the correlation coefficient. As shown in Table 2, the square root values of AVE of each construct were higher than their correlations with other factors [62]. Thus, discriminant validity was supported. Finally, we confirmed that the research model provided the best representation by comparing different models. As shown in Table 4, the three-factor model of AEC, psychological contract breach and cyberloafing had an excellent fit to the data (*χ*^2^/d*f* = 2.619 < 5; CFI = 0.924 > 0.900; IFI = 0.925 > 0.900; TLI = 0.910 > 0.900; RMSEA = 0.076 < 0.080) [63]. Thus, the model fit the data well. In addition, we examined the VIF value to detect whether multicollinearity threatened the research model. As shown in Table 3, the VIF values of independent variables are 1.367 and 1.375 respectively, which are within the threshold of 10 [64]. This indicated that there was no severe multicollinearity in this study. Therefore, the model was suitable for testing the research hypotheses.

**Table 3:** Measurement model

Construct	Item	Factor loadings	CR	AVE	VIF
AEC	AEC1	0.706	0.759	0.513	1.367
	AEC2	0.728			
	AEC3	0.714			
Psychological contract breach (PC)	PC1	0.803	0.827	0.545	1.375
	PC2	0.716			
	PC3	0.692			
	PC4	0.736			
Cyberloafing (CY)	CY1	0.771	0.901	0.507	
	CY2	0.768			
	CY3	0.658			
	CY4	0.814			
	CY5	0.743			
	CY6	0.789			
	CY7	0.622			
	CY8	0.586			
	CY9	0.614			

Note(s): Abbreviations: AVE, average variance extracted; CR, composite reliability; AEC, After-hours electronic communication; PC, psychological contract breach; CY, cyberloafing

**Table 4:** Comparison of competition models

Models	*χ* ^2^	d*f*	*χ*^2^/d*f*	CFI	IFI	TLI	RMSEA
Three-factor model	264.549	101	2.619	0.924	0.925	0.910	0.076
Two-factor model 1	366.576	103	3.559	0.878	0.879	0.858	0.095
Two-factor model 2	434.715	103	4.221	0.847	0.848	0.821	0.107
One-factor model	516.234	104	4.964	0.81	0.811	0.780	0.119

Note(s): The one-factor model is AEC + psychological contract breach + cyberloafing; two-factor model 1 is AEC + psychological contract breach and cyberloafing; two-factor model 2 is AEC and psychological contract breach + cyberloafing; the three-factor model is AEC, psychological contract breach and cyberloafing

### Testing of hypotheses

**Fig. 2:**
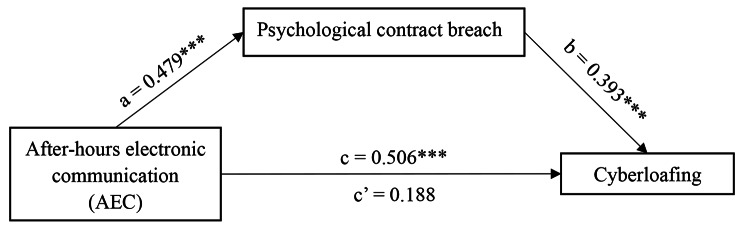
Path coefficients

As shown in Table 5, the findings of bootstrapping analysis demonstrated that AEC had a significant positive effect on nurses’ cyberloafing (*β* = 0.506; *p* < 0.001) and psychological contract breach (*β* = 0.479; *p* < 0.001), supporting H1 and H2. When both AEC and psychological contract breach were considered, the effect of psychological contract breach on nurses’ cyberloafing was 0.393 (*p* < 0.001), supporting H3. And, AEC still had a significant positive effect on nurses’ cyberloafing (*β* = 0.318; *p* < 0.001), indicating that psychological contract breach played a mediating role between AEC and cyberloafing. As shown in Table 6, the indirect effect of AEC on cyberloafing through psychological contract breach was 0.188 (95% bootstrap CI = [0.119, 0.273]). The indirect effect of psychological contract breach was significant at a 95% level since the 95% bootstrap CI did not contain zero, which proved that psychological contract breach was significant mediator of the effect of AEC on nurses’ cyberloafing. Therefore, AEC was significantly indirectly correlated with nurses’ cyberloafing via psychological contract breach. And the mediating effect accounted for 37.15% of the total effect. Thus, the data were supportive of H4. Figure 2 reflects the path coefficients in the study.

**Table 5:** Mediating effect analysis

Variables	Cyberloafing			Psychological contract breach			Cyberloafing		
	*β*	SE	*p*	*β*	SE	*p*	*β*	SE	*p*
Gender	0.155	0.183	0.396	0.237	0.205	0.249	0.062	0.165	0.707
Age	0.227	0.124	0.068	0.152	0.140	0.279	0.168	0.112	0.135
Clinical tenure	-0.176	0.070	0.012	-0.062	0.079	0.433	-0.152	0.063	0.017
Education level	0.240	0.076	0.002	0.169	0.085	0.050	0.173	0.069	0.012
AEC	0.506	0.046	< 0.001	0.479	0.051	< 0.001	0.318	0.047	< 0.001
Psychological contract breach							0.393	0.048	< 0.001
R^2^	0.370			0.273			0.493		
F	32.353			20.683			44.483		
P	< 0.001			< 0.001			< 0.001		

Note(s): SE = Standard error

**Table 6:** Bootstrap based mediation effect test

Effect	Mediation path	β	Boot SE	Boot LLCI	Boot ULCI
Indirect effect	AEC → Psychological contract breach → Cyberloafing	0.188	0.039	0.119	0.273

Note(s): Bootstrap 95% confidence intervals for indirect effects. SE = Standard error, LLCL = Lower limit confidence interval, UCL = Upper limit confidence interval

## Discussion

### Interpreting the findings

We identified that both AEC and psychological contract breach have direct positive impacts on nurses’ cyberloafing, and psychological contract breach mediated the relationship between AEC and cyberloafing. First, this study revealed that AEC directly leads to nurses’ cyberloafing, which is consistent with the previous finding that AEC increases the likelihood of counterproductive behavior [52]. AEC failings to compensate nurses for the extra time, energy, and other resources they paid for working activities in nonworking context. Nurses respond to AEC by reducing their commitment to work. Our study also reinforces the previous view that cyberloafing is a way for employees to cope with AEC [26].

Second, AEC is positively related to psychological contract breach. Previous studies have found that when tasks are illegitimate, employees will have a sense of unfairness and psychological contracts will be affected [65]. AEC requires nurses to respond to organizational assignments during nonworking hours without reward. The perceived unfairness caused by AEC will lead to nurses’ psychological contract breach. The research of Wiechers et al. also confirmed that the sense of unfairness is an important factor leading to employees’ psychological contract breach [45].

Third, this study found that psychological contract breach is positively related to cyberloafing. Psychological contract breach represents nurses’ perception that their organization has not fulfilled its obligations. When psychological contract breaks, identification with the organization decreases and nurses are more likely to engage in cyberloafing. Many studies have confirmed from different perspectives that psychological contract breach is associated with negative career-related behavior [46, 66].

Additionally, this study found that psychological contract breach plays a mediating role in the relationship between AEC and nurses’ cyberloafing, which is consistent with previous studies indicating that AEC (the phenomenon of effort-reward imbalance) has negative effects on employees through psychological contract breach [67, 68]. With the rapid development of modern society, leisure activities have gradually become an important part of people’s well-being [69]. However, AEC reduces the time for nurses to carry out leisure activities and leads to anxiety and other reactions [70]. Negative experiences in nonwork field caused by AEC will affect employees’ performance in work field through psychological contract breach. The result was similar to that of Sahay who also argued that psychological factors are the bridge between work field and nonwork field [19].

### Theoretical implications

The novelty of this research is derived from its research limitations and practical implications.

First, one major theoretical contribution of the current study is that we confirmed the spillover effect of AEC on nurses’ cyberloafing occurs via psychological contract breach. Previous studies on the antecedent variables of cyberloafing have mostly focused on organizations [1], which are all in the workplace. Although through AEC, nurses have to deal with work-related matters, it occurs in the non-workplace. The current study extends past research by investigating the spillover effect from the non-workplace to the workplace and enriches the research on antecedent variables of inducing nurses’ cyberloafing.

Second, this study reveals the impact of AEC on negative extra-role behaviors of employees. Although previous studies have discussed the outcome variables of AEC, they mainly focus on its influence on employee health, family, emotions, and happiness, etc. This study finds that AEC has a positive effect on nurses’ cyberloafing, which extended our knowledge of the negative outcome variables of AEC.

Third, this study echoes the call for more research on the relationship between organizational communication norms and cyberloafing [1, 13]. The results from this study indicate that AEC, a nonnormative form of organization communication, can lead to the psychological contract breach and thus result in nurses’ cyberloafing.

Finally, our study extents the research scope of AEC and nurses’ cyberloafing. Previous studies have forced on employees in common industries. This study takes nurses as the research object and focuses on the cyberloafing of nurses. Expanding the previous research scope.

### Practical implications

These findings also have important practical implications. The identified relationships between the variables provide a guide for healthcare organizations to reduce or manage inappropriate telework arrangements as well as strengthen nurses’ psychological contracts. The increasing aging and people’s increasing emphasis on medical care have led to the continuous expansion of medical institutions and the urgent need for nurses. Many nurses have to be ‘always online’ to respond to the needs of the organization, even during non-working time. However, this study found that frequent electronic communication during non-working hours would lead to nurses’ psychological contract breach, and thus induce cyberloafing. This reminds the organization to grasp the frequency of AEC, strengthen the control and intervention on the negative impact of AEC.

First, the organization should attach importance to creating a positive, cherish the time of the working atmosphere. The organization needs to set clear rules and regulations and corresponding reward and punishment mechanisms, strengthen nurses’ awareness of time management, and reduce the tolerance of cyberloafing within the organization. And healthcare organizations should supplement nurses in time to avoid staff shortage. The salary of nurses can be increased to attract more people to the nursing profession.

Second, organizations should also maintain the psychological contracts through effective communication channels. The organization should timely pay attention to the nurses who have negative emotions due to excessive work pressure and immediately dredge bad mood.

Finally, for nurses, they also need to take some measures to avoid falling into the vicious cycle of dealing with work in non-working time and killing time in working time. Nurses should cultivate efficient work habits to reduce unnecessary AEC.

### Limitations and future studies

It is rational to have several limiting factors in the article. First, our empirical results rely entirely on cross-sectional data. While our findings provide valuable information for the study of the relationship between AEC and nurses’ cyberloafing, we acknowledge that we did not infer the causality of the associations between them. Future studies are needed to consider the possibility of causation or reverse causation between AEC and cyberloafing. To this extent, studies with stronger designs are needed. Longitudinal study design is a serviceable instrument for examining cause-effect relationships. In addition, another reason why we call for using longitudinal design is that nurses’ responses may vary over time, which can lead to different estimates of measurements at a certain point in time and self-reports data. Therefore, when exploring the relationship between AEC and cyberloafing, a longitudinal study design is valuable (as it can reflect the change of nurses’ responses over time), and a cross-sectional study is also indispensable (as it can reflect nurses’ current attitudes). Together, they can achieve a comprehensive understanding of the relationship between AEC and nurses’ cyberloafing.

Second, relying entirely on self-report measures for our samples leads to an inescapable problem, that is, our research results are affected by potential subjective bias and social expectations bias among participants. In other words, the response bias of the investigated nurses in this study could affect the exactitude of the assessment. Because they are reluctant to let others (especially organizations) know that they are dealing with nonwork affairs during work hours, nurses may likely report similar low-level cyberloafing results to show that they work attentively. In order to minimize the influence of external standard answers, we repeatedly assured that the results were absolutely confidential, and the answers were no false or correct. Despite all this, the biases were still difficult to eliminate. In future study, other methods can be considered to obtain more objective data, such as observation method, multi-data source synthesis method and so on. Moreover, in a more extensive context, different social environments lead to different behaviors [71, 72]. All participants were recruited from China. Since healthcare systems vary from country and country, this may raise concerns about external effectiveness. Therefore, the results of this study may not apply to nurses in all countries. Future studies should replicate our research in other countries and cultural contexts to enhance its generalizability.

Third, previous studies have not reached a consensus on whether gender and age are significantly related to cyberloafing [7, 73]. Our study found that gender and age were not significantly correlated with cyberloafing. In terms of age and cyberloafing, it may be because the availability of network resources weakens the influence of age on cyberloafing. In terms of gender and cyberloafing, the results of this study may be limited by the sample. Due to the nature of the data collection environment in this study (i.e., there are far more female nurses than male nurses in China), the current sample was overwhelmingly female nurses. This may have had an important impact on the results. Therefore, it is necessary to explore the influence of gender on cyberloafing in different contexts with large male nurses in future studies.

Finally, we only focused on the influence of AEC on nurses’ neglect behavior (i.e., cyberloafing). According to employee behavioral options (namely exit, voice, loyalty and neglect, EVLN model), employees have four types of behavioral choices when experience job dissatisfaction [74]. In the future, the influence of AEC on nurses’ behaviors can be studied in detail under the framework of EVLN model.

With the continuous development of technology, the background of AEC and cyberloafing is process of expanding. AEC and cyberloafing are receiving more and more attention around the world. AEC and cyberloafing are two products derived from ICT, which makes the boundary between work and private life invisible. In terms of AEC, ICT usage is a symbol of the all-weather work culture. Nurses are compelled to keep in touch both day and night. In terms of cyberloafing, the use of ICT allows nurses to access to the internet from almost everywhere without control. As a result, with the rapid progress of technology, it is worth thinking deeply about how to make good use of ICT to improve nurses’ job satisfaction and family happiness.

## Conclusions

This research represents an initial attempt to explore the influencing factors of nurses’ cyberloafing from the non-workplace as opposed to the workplace. In particular, we highlight the spillover process of psychological contract breach between AEC and nurses’ cyberloafing.

## Data Availability

The datasets used and/or analysed during the current study are available from the corresponding author on reasonable request.

## Abbreviations

AEC: After-hours electronic communication
